# A predictive model of genital warts preventive behaviors among women in the south of Iran: application of health belief model

**DOI:** 10.1186/s12905-022-01649-6

**Published:** 2022-03-08

**Authors:** Saeideh Shahsavari, Azin Alavi, Parisa Razmjoue, Shokrollah Mohseni, Vahid Ranae, Zahra Hosseini, Sakineh dadipoor

**Affiliations:** 1 Mother and Child Welfare Research Center, Faculty of Nursing and Midwifery, Hormozgan University of Medical Sciences, Bandar Abbas, Iran; 2 Department of Obstetrics and Gynecology, Maternal-Fetal Medicine Research Center, School of Medicine, Shiraz University of Medical Sciences, Shiraz, Iran; 3 Infectious and Tropical Diseases Research Center, Hormozgan Health Institute, Hormozgan University of Medical Sciences, Bandar Abbas, Iran; 4 Student Research Committee, Hormozgan University of Medical Sciences, Bandar Abbas, Iran; 5 Tobacco and Health Research Center, Hormozgan University of Medical Sciences, Bandar Abbas, Iran; 6 Social Determinants in Health Promotion Research Center, Hormozgan Health Institute, Hormozgan University of Medical Sciences, Bandar Abbas, Iran

**Keywords:** Genital warts, Women, Human papillomavirus, Health belief model

## Abstract

**Background:**

Genital wart (GW) is known as an infectious disease. Besides the infection, it is associated with a higher risk of cervical neoplasia and cancer in the infected population. The present research aimed to explore the predictors of GW preventive behaviors based on the health belief model (HBM).

**Methods:**

The present analytical and cross-sectional research was conducted in 2019 among 720 women between 15 and 49 years of age in Bandar Abbas in the south of Iran. The sample was selected in a multi-stratified clustering method. The participants responded to a reliable and valid researcher-made questionnaire which explored demographic information, knowledge-related items and the model constructs. A multivariate linear regression analysis was run to determine the predictors of adopting GW preventive behaviors. A path analysis was also run to test the direct and indirect effects of the model constructs on the dependent variable.

**Results:**

The mean and standard deviation of participants’ age was 30.43 ± 8.697 years. As Pearson’s correlation coefficients showed, knowledge (r = 0.197, *p* < 0.001), perceived susceptibility (r = 0.434, *p* < 0.001), severity (r = 0.463, *p* < 0.001) and self-efficacy (r = 0.434, *p* < 0.001) were significantly correlated with the adoption of GWs preventive behaviors. Multiple linear regression analysis showed that self-efficacy (B = − 0.010, *p* < 0.001), perceived susceptibility (B = 0.070, *p* < 0.001) and severity (B = 0.078, *p* < 0.001) were the predictors of GW preventive behaviors. Path analysis showed that perceived susceptibility, severity and self-efficacy directly affected healthy behaviors while perceived benefits and barriers indirectly affected the preventive behaviors.

**Conclusions:**

The present findings help to promote knowledge of the predictors of GW preventive behaviors. HBM can be a useful theoretical framework to evaluate the preventive behavior of the disease and help to reduce the rate of sexually-transmitted infections including GW.

**Supplementary Information:**

The online version contains supplementary material available at 10.1186/s12905-022-01649-6.

## Background

Genital warts are known as a clinical form of human papillomavirus (HPV) infection because they are injuries in the form of single or multiple papules in the area of vulva, perineum, anus, vagina or cervix. They are typically associated with HPV6 and HPV11, and also with other viruses such as HPV2, 40, 42, 43 and 54 [1].

A body of related research showed that about 6.2 million new forms of GW occur annually among people between 14 and 44 years of age [2]. The prevalence and incidence rate of GW in Italian women was reported to be 3.8 and 3.0 per 10,000 people and 3.39% among women in Philippine [1]. GW is also prevalent among Iranian women [3–5]. Shafaghi et al. explored the prevalence of GW in 851 Iranian women, and reported 265 cases afflicted with 19 different types of HPV including GW [6].

GW is highly infectious. About 65% of individuals with an infected sex partner get afflicted with GW within 3–8 months [7]. Besides their highly infectious property, GW is associated with a high risk of cervical intraepithelial neoplasia and cancer in women with a history of GW [8]. Moreover, GW tremendously affects the quality of a patient’s life [1]. It increases mental stress to a great extent. The cost imposed on the health system annually is estimated at about 200 million dollars [9]. There is yet no HPV vaccination in Iran and culturally having a sex affair is only accepted for married women. Thus, administering a test for sexually transmitted infections is a challenge for most women [10]. Evidently, trying to prevent this infection can be the best strategy in this geographical zone. A study found that preventing high-risk behaviors associated with GW can be effective in controlling this disease [8]. Moreover, preventing risky behaviors can largely depend on raising awareness and changing attitudes and beliefs [11].

A body of research in Iran reported a low level of women’s and girls’ knowledge of HPV and GWs [4, 12, 13]. In fact, only 8% of patients with GW showed to wear condoms [14]. However, in Hong Kong, 65% of patients infected with HPV used condoms [15]. These facts and figures proved the absence of GW preventive behaviors in Iranian population. A study in Iran showed that raising awareness of GW can be effective in reducing the epidemic [16]. Probably, awareness-raising affects attitude, increases one’s susceptibility to infection and increases the perceived risk. It, thus, has a preventive role in affliction with GW.

When GW epidemic has certain adverse effects, seemingly the first step to control the disease, as in preventing many other health threats, is to adopt preventive and protective behaviors to impede the occurrence of GW and prevent the cancer induced by some high-risk genotypes [17]. Health education models can be a great help in this regard. Among them is the health belief model (HBM) which explores the association between beliefs and behavior and is more involved in the prevention of diseases. The existing body of research shows that HBM is a useful framework for exploring health education topics including sex issues [18].

### The health belief model

**Fig. 1:**
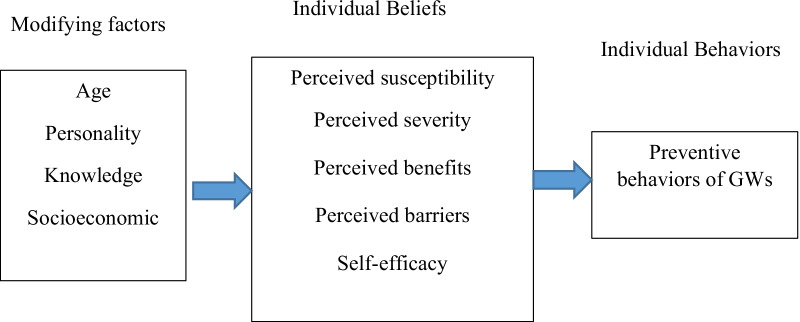
Theoretical model (health belief model) used preventive behaviors of GWs

A major educational model within the health education domain is health belief model (HBM), which plays a significant role in adopting preventive behaviors. This model concerns how our perception can motivate us to behave in a certain way. Based on HBM, to adopt preventive behaviors, people should first perceive susceptibility to GW. Then, they need to perceive the severity of the disease and its adverse physical, mental, social and economic effects. People should also perceive the cues for action from their internal or external environment and prefer the perceived benefits of the preventive program to the perceived barriers (physical, economical or mental barriers to preventive behaviors). This would make them capable of showing preventive behaviors and is known as perceived self-efficacy. They will finally be capable of adopting GW preventive behaviors [19, 20] (Fig. 1).

Previous studies primarily explored the prevalence and incidence of GW among women [1, 21], the major predictors of using HPV vaccination [22, 23], knowledge [12, 24], relationship between demographic information and GW [16] or high-risk populations with multiple sex partners or those with sexually transmitted diseases [25]. Besides, due to the dearth of regional information about GW infection [26], it needs to be explored separately in different populations. As a review of the related literature shows, the present research is pioneering in determining the predictors of GWs preventive behaviors based on HBM among women in the south of Iran. The present researchers believe that recognizing the predictors of GW preventive behaviors and providing this information to healthcare policy-makers are the major steps toward reducing the rate of the disease and its prevalence.

## Methods

### Research design and population

The present analytical and cross-sectional study was conducted in 2019 in Bandar Abbas in Hormozgan Province in the south of Iran. The city is of 27.19 latitude and 56.28 longitude, and is located at the height of 9 m above the sea level. Bandar Abbas has a population of 352,173, which makes it the largest and most populated city in Hormozgan Province. The research population comprised all women between 15 and 49 years of age selected from comprehensive healthcare centers. The inclusion criteria were being female, being between 15 and 49 years old, not being infected with sexually transmitted diseases, having no history of sexual diseases, sharing Iranian nationality and giving an informed consent to take part in the research. The exclusion criterion was failure to hand in complete questionnaires.

### Sample size and selection

As one purpose of this study was to determine the score of GW preventive behavior, the following formula was used to estimate the sample size: $n = \frac{{z_{{1 - \frac{\alpha }{2}}}^{2} \delta^{2} }}{{d^{2} }}$. In the related studies, for instance, Namdar et al. [27] the score of cervical cancer preventive behavior was 1.9 ± 1.64. Thus, if $z_{{1 - \frac{\alpha }{2}}} = 1.96$, $\delta = 1.64$ and $d = 0.15$, the above-mentioned formula gives us a sample of 460 participants. The sampling method was clustering. Thus, to estimate the final sample size, a design effect of 1.6 was considered. The final sample size was 420 × 1.6 = 760.

**Fig. 2:**
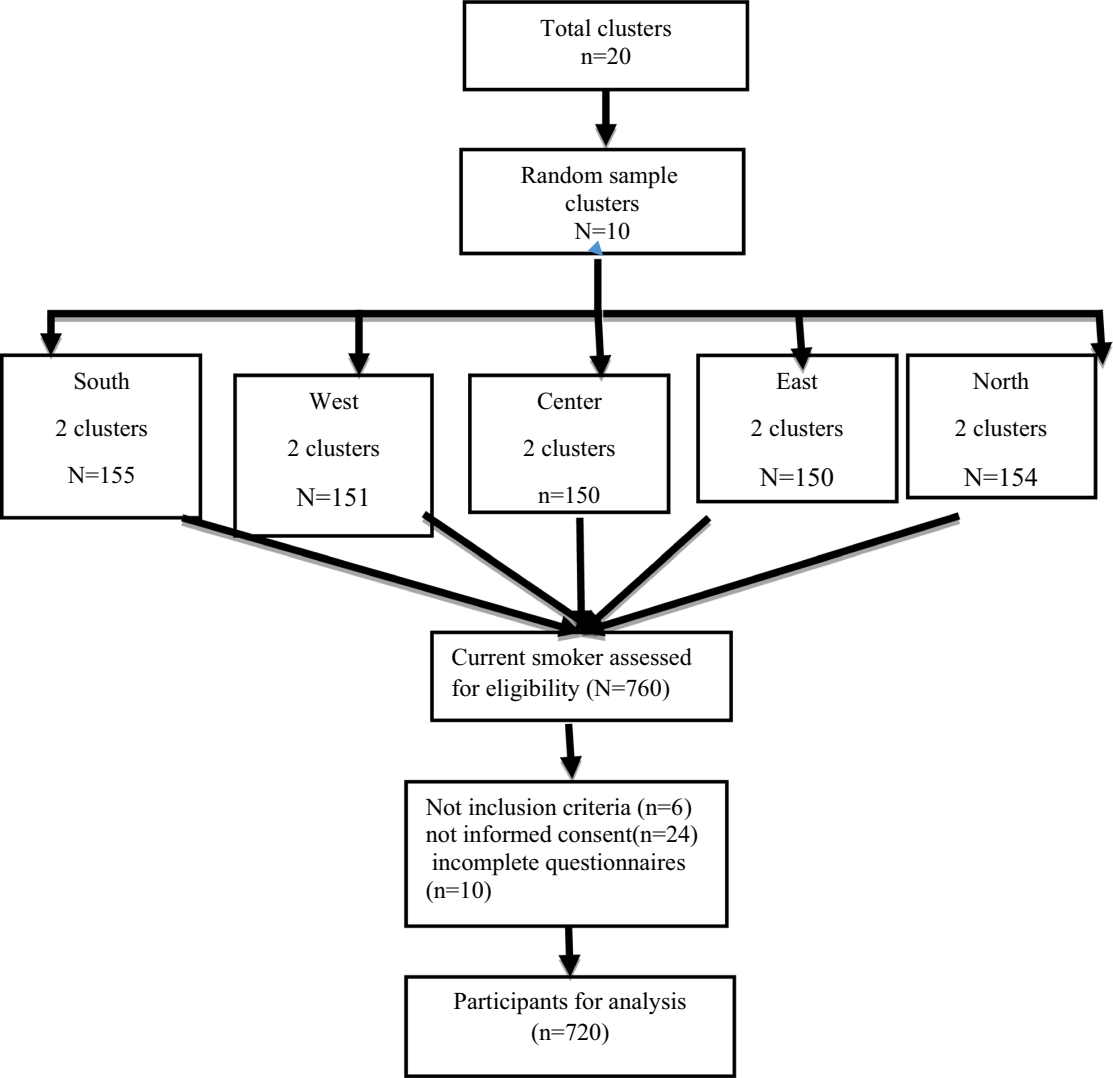
Flowchart of the sampling procedure

The participants were selected in a multi-stratified clustering method and through simple randomization. To this aim, from 20 comprehensive healthcare centers in Bandar Abbas, 15 clusters were selected and then the quota for each cluster was determined according to the population covered. To select participants from each cluster, a systematic randomization method (a list of households) was used. Then, from each cluster, a region was selected; from each region a particular street was selected, and then from each street an alley was selected to be visited. The clusters and the relevant households were visited and enlisted (including all family members living together) and then one was randomly selected which met the inclusion criteria and was visited for data collection (Fig. 2).

### Data collection

The data were collected using a researcher-made questionnaire completed upon visiting the respondents’ house. The questionnaire completion took about 10 min. Literate women completed the questionnaires at home at their convenience and returned the completed questionnaires to the researcher later on. For the illiterate, the questions were read out loud by the researcher with minimal bias. If a participant who met the inclusion criteria was not present at home, the researcher revisited the house at a later time. If the researcher failed to meet the participant three times, she continued collecting data from other families so as to obtain as much information as required.

### Measurement procedure

The self-administered questionnaire contained questions about: (a) demographic information, (b) knowledge of GWs (symptoms, ways of transmission, risk factors), and (c) HBM constructs.

The three parts of questionnaire will be introduced below with their content.

It is noteworthy that Part 1 and Part 2 are added (are among the moderating factors now included in the HBM model).

#### Part 1

This part included the participants’ demographic information including their age (in years). The participants were, thus, divided into 4 age categories: < 19, 20–29, 30–39 and > 40, marital status (married, divorced, widowed), presence of another sex partner than husband (yes/no), educational level (illiterate, below diploma, diploma, academic), occupation (not working, working outside home), socioeconomic level (lower, middle, upper) and the insurance coverage (yes/no). Socioeconomic status (SES) was based on the distribution of household crowing index (the person per room ratio). The decreasing crowdedness levels were categorized as upper, middle or lower SES (crowding index < 1, 1–2, and > 3 people per room) [28, 29].

#### Part 2

This part explored the participants’ knowledge of GW with 24 questions each with three choices (true, false, don’t know). A true answer received a score of 1 while a false or don’t know answer received 0. These questions addressed aspects of knowledge including the ways of transmitting infection, symptoms and ways of preventing GWs. The reliability of the knowledge part was already substantiated in a study by Farshbaf et al. among female residents of Tabriz (a city in the northeast of Iran). The reliability coefficient was estimated at 82% [29].

#### Part 3

This part explored the HBM constructs and included 6 sub-scales as introduced below.

*Perceived susceptibility* It contained 7 items including “I have higher chances of affliction with GW in the next few years” and “Most probably I will be afflicted with GW throughout my life”.

*Perceived severity* It contained 5 items. An instance is “I have been facing GW-related challenges and issues for years”.

*Perceived benefits* The timely diagnosis of GW to prevent the severe symptoms was evaluated with 7 items. Here is an instance: “An early administration of the Pap test and an early diagnosis of GW prevent extra medical costs”.

*Perceived barriers* The adoption of GW preventive behaviors was assessed through 10 items such as: “The healthcare center is crowded and they do not have enough time to examine everyone. So, I prefer not to go”.

*Self-efficacy* Here 6 items were used to assess an individual’s capability of showing GWs preventive behaviors. Instances of items are: “I am sure I can obey sexual health rules to reduce the rate of affliction with GWs”.

### Preventive behaviors of GW

Healthy behavior was assessed along 4 items including: “I use condoms in every sex relationship”.

All items of the sub-scales were rated on a 5-point Likert scale: (1) Strongly agree, (2) Agree, (3) Neutral, (4) Disagree, (5) Strongly disagree. Each sub-scale was scored separately and not altogether as a total score. The sub-scale scores were calculated and reported for each participant. Higher scores showed stronger feelings about a construct. All subscales had positive responses about the target behavior except for perceived barriers which was negatively associated.

### Data quality assurance

To develop the questionnaire, at first, a library research was done about the topic of interest. Thus, a first draft of the questionnaire was developed based on HBM. To substantiate its content validity, the questionnaire was provided to a panel of 5 experts in health education and 5 gynecologists. Their comments were used to revise the questionnaire.

To test the reliability of the questionnaire, a test–retest method was used. To this aim, the questionnaire was provided at a 2-week’s interval to 30 individuals of similar conditions to the main participants. Then, each item in the first test was compared to the retest. If the correlation coefficient between the test and retest in each scale and sub-scale was above 0.7, the reliability was confirmed. To check the consistency of the test and retest, ICC was used, which was estimated at 0.83 and was confirmed.

### Ethical considerations

To collect the required data, a formal permission was gained from the university deputy of research for visiting a sample of healthcare centers. Upon visiting the place, the researcher introduced herself completely and revealed the purpose of research for the women. Then, a written letter of consent was provided for the participants to sign. It contained all details of the research. The voluntary participation of all participants was warranted. They were asked not to reveal their identity in the questionnaires. Thus, they were assured of the confidentiality of the information they provided. This research was approved by Hormozgan University of medical sciences (#IR.HUMS.REC.1398.267).

### Data analysis

The quantitative variables were measured through descriptive statistics (standard deviation, maximum, minimum, mean, range) and the qualitative variables were described as frequencies and percentages. Pearson correlation coefficient was used to map the correlation matrix of the model constructs. Multivariate linear regression analysis was used to test a correlation between the model constructs, knowledge and behavior. A test of the association was used to explore the interrelationships among the model constructs, knowledge and behavior. Path analysis was run to test the direct and indirect effect of the model constructs on the dependent variable. To this aim, AMOS was used. The rest of analyses were conducted in SPSS21 and the significance level was set at *p* < 0.05.

## Results

### Sample characteristics

Among the 760 questionnaires distributed, 720 were returned. Overall, 40 women were excluded from the study for not signing the informed letter of consent, missing information for a crucial item or only answering the demographic questions. The data acquired from 720 participants were finally statistically analyzed. The mean ± SD of age, age of marriage and age of the first sex affair was 30.43 ± 8.697, 21.08 ± 4.13 and 20.78 ± 4.15 years, respectively. The majority of participants belonged to the 20–29 year age group (40%), were married (85.6%), had an average SES (76.4%), an education level below diploma (36.4%), and were housewives (70.3%). The other detailed demographic information is summarized in Table 1.

**Table 1:** Research participants’ demographic information (n = 720)

Variable	Categories	Frequency (n)	Percent (%)
Age	< 19	64	8.9
	20–29	288	40.0
	30–39	228	31.7
	> 40	140	19.4
Multiple sex partners	Yes	90	12.5
	No	630	87.5
Educational level	Illiterate	62	8.6
	Below diploma	262	36.4
	Diploma	212	29.4
	Academic	184	25.6
Economic status	Upper	76	10.6
	Middle	550	76.4
	Lower	94	13.1
Marital status	Married	616	85.6
	Divorced	72	10.0
	Widow	32	4.4
Working status	Working outside home	214	29.7
	Not working	506	70.3
Under insurance	Yes	534	74.2
	No	186	25.8

Table 2 summarizes the mean, median and standard deviation of knowledge, HBM constructs and preventive behaviors. As it can be seen, the scores for perceived benefits and severity were below average while other constructs were about the average score.

**Table 2:** Mean ± standard deviation of knowledge, HBM constructs and GWs preventive behaviors (n = 720)

Variable	Mean ± SD	Median	Score range	The percentage of score obtained from the maximal score
Knowledge	14.03 ± 4.29	14.00	0–22	63.77
Perceived susceptibility	18.18 ± 3.68	18.00	6–27	67.33
Perceived severity	17.21 ± 3.19	18.00	5–25	68.84
Perceived benefits	27.63 ± 3.08	28.00	18–35	78.94
Perceived barriers	29.55 ± 4.51	29.00	15–50	59.1
Self-efficacy	22.50 ± 3.20	22.00	10–30	75
Preventive behaviors of GWs	3.06 ± .807	3.00	0–4	76.5

The correlation matrix of the HBM constructs with each other and with knowledge can be viewed in Table 3. As the results showed, statistically significant correlations were found between all constructs except for perceived benefits and barriers with GW preventive behaviors (Table 3).

**Table 3:** Pearson correlation coefficient of the HBM constructs and GWs preventive behaviors

Variables	Knowledge r (p)	Perceived susceptibility r (p)	Perceived severity r (p)	Perceived benefits r (p)	Perceived barriers r (p)	Self-efficacy r (p)	Preventive behaviors of GWs
Knowledge r (p)	1						
Perceived susceptibility	0.149 (< 0.001)	1					
Perceived severity	0.254 (< 0.001)	0.390 (< 0.001)	1				
Perceived benefits	0.307 (< 0.001)	0.057 (0.126)	0.061(102)	1			
Perceived barriers	− 0.326 (< 0.001)	0.173 (< 0.001)	0.050 (0.179)	− 0.178 (< 0.001)	1		
Self-efficacy	210 (< 0.001)	0.020 (0.588)	0.099 (0.008)	0.456 (< 0.001)	− 0.143 (< 0.001)	1	
Preventive behaviors of GWs	0.197 (< 0.001)	0.434 (< 0.001)	0.463 (< 0.001)	0.027 (< 0.281	− 0.034 (0.356)	0.434 (< 0.001)	1

A multivariate linear regression analysis was used to test the correlation between HBM constructs and the dependent variable (preventive behavior). As tabulated, perceived susceptibility, severity and self-efficacy showed statistically significant correlations; however, perceived barriers and benefits had no statistically significant correlation with GW preventive behaviors. Overall, this model managed to explain 45% of the variance in dependent variable. Among all constructs, self-efficacy showed to be the strongest predictor of the target healthy behavior (B = 0.098) (Table 4).

**Table 4:** Multivariate regression analysis of HBM constructs and GWs preventive behaviors

Variable	Unstandardized coefficients		t	Sig.	95.0% confidence interval		R square
	B	Std. error			Lower bound	Upper bound	
(Constant)	− 1.448	0.306	− 4.728	0.000	− 2.049	− 0.846	0.449
Perceived susceptibility	0.070	0.007	10.325	0.000	0.057	0.083	
Perceived severity	0.078	0.008	9.976	0.000	0.063	0.094	
Perceived benefits	0.003	0.008	351	0.726	− 0.014	0.020	
Perceived barriers	− 0.010	0.005	− 1.922	0.055	− 0.021	0.000	
Self-efficacy	0.098	0.008	12.387	0.000	0.083	0.114	

**Fig. 3:**
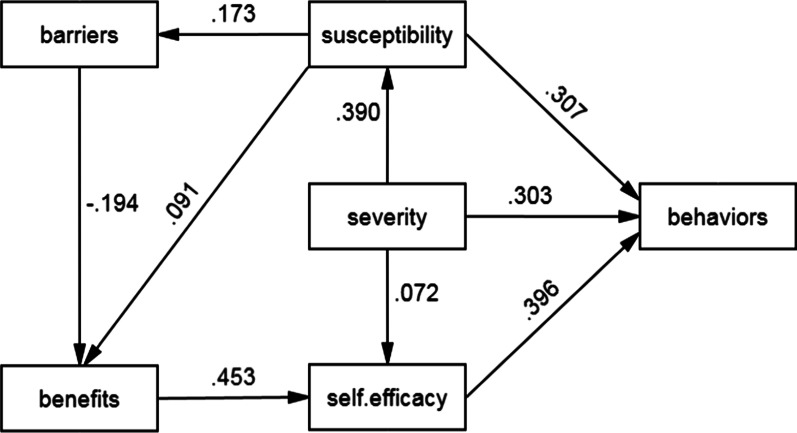
The relationship between model constructs and behavior

Path analysis: The final path analysis showed that self-efficacy (B = 0.396, *p* < 0.001), perceived susceptibility (B = 0.307, *p* < 0.001), and perceived severity (B = 0.303, *p* = 0.034) directly affected GWs preventive behaviors. Perceived benefits and barriers had no direct effect on healthy behavior. Yet, these two variables indirectly affected the target behavior (Fig. 3).

The direct, indirect and total effects were tested too and perceived severity with a total effect of 0.455 showed to have the strongest effect. Next ranked self-efficacy, perceived susceptibility, benefits and barriers, respectively (Table 5). Overall, three variables together, self-efficacy, perceived severity and susceptibility explained 44% of the total variance in the target behavior. Two variables, perceived benefits and severity accounted for 21% of the variance in self-efficacy (Table 6). According to Table 7, the overall goodness of fit indices attest to the model fitness.

**Table 5:** Direct and indirect effects of HBM constructs on GWs preventive behaviors in path analysis

Variable name	Direct effects	Indirect effects	Total effects
Severity	0.303	0.152	0.455
Self-efficacy	0.396	0.000	0.396
Susceptibility	0.307	− 0.010	0.317
Benefits	0.000	0.179	0.179
Barriers	0.000	− 0.035	− 0.035

**Table 6:** Path coefficients and the variance in HBM constructs explained

Path			β	S.E	C.R	*p*	R^2^
Severity	→	Susceptibility	0.390	0.040	11.370	< 0.001	0.152
Susceptibility	→	Barriers	0.173	0.045	4.721	< 0.001	0.030
Susceptibility	→	Benefits	0.091	0.031	2.444	0.015	0.040
Barriers	→	Benefits	− 0.194	0.025	− 5.232	< 0.001	
Benefits	→	Self-efficacy	0.453	0.034	13.662	< 0.001	0.211
Severity	→	Self-efficacy	0.072	0.033	2.165	0.030	
Severity	→	Behaviors	0.303	0.008	10.036	< 0.001	0.448
Susceptibility	→	Behaviors	0.307	0.007	10.181	< 0.001	
Self-efficacy	→	Behaviors	0.396	0.007	14.244	< 0.001

**Table 7:** Goodness of fit indices for the predictive model of GWs preventive behaviors

**Χ**^**2**^	DF	**Χ**^**2**^/DF	GFI	AGFI	NFI	CFI	IFI	RMSEA	RMR
8.985	6	1.497	0.996	0.986	0.988	0.996	0.990	0.026	0.260

## Discussion

The present research explored the adoption of GW preventive behaviors based on HBM constructs.

To our best knowledge, so far, no similar research has been conducted using HBM or any similar model for predicting GW preventive healthy behaviors. Thus, the present research is pioneering in using HBM to predict the determinants of adopting GWs healthy behaviors.

As the results showed, knowledge, perceived susceptibility, severity and self-efficacy had statistically significant correlations with the above-mentioned healthy behavior. Among the constructs, self-efficacy was the strongest predictor of adopting GW preventive behaviors.

The present findings revealed a statistically significant correlation between knowledge and adoption of GW preventive behaviors. Similarly, some other research showed that a high level of knowledge could predict HPV vaccination 7.97 times as much as a low level of knowledge [30]. In their research, Sari and Syahrul maintained that 76% of women who received the HPV vaccine enjoyed a high level of knowledge. However, women who did not receive the vaccine had a moderate or low level of knowledge [31]. Contrary to the present findings, a number of other studies found no statistically significant correlation between knowledge and adoption of healthy behaviors [22, 32]. Even in another study, insufficient knowledge showed to induce a better acceptance of the HPV vaccine [33]. Thus, knowledge can be seen as critically involved in forming attitude and behaviors. Adoption of new behaviors is easier when it is based on a correct knowledge, high awareness and positive attitude. Some research revealed that knowledge indirectly moderated the effect of attitude on the intention of HPV vaccination [34]. Undoubtedly, awareness-raising is the most influential factor in raising sex awareness, and awareness-raising through mass media can prevent the prevalence of sexually transmitted diseases among people. In Iran, contrary to other developing countries, sex education has been conspicuously absent, and this is mostly due to the dominant culture and traditions. Absence of any sex education for long can lead to a low overall level of sex knowledge in society and can be associated with higher risks of sexually transmitted diseases such as GW. Iranian women’s knowledge of sex issues is truly limited. Thus, sex education is essential for Iranian women [10]. It is suggested that sex awareness be raised in both society and families so that everyone can benefit from this knowledge.

The present findings showed that the effect of perceived susceptibility on GW preventive behaviors was statistically significant. This finding points to the fact that women who perceive themselves at a higher risk of GW adopt more protective behaviors. In other words, if their perceived susceptibility is low, their susceptibility to and precision in adopting healthy protective behaviors may be low too. Resentstock (1982) in Ningrum (2016) maintained that those who perceive the adverse effects of a disease more than others show more tendency to medical preventive services [35]. Similarly, another study showed that women who perceived themselves more susceptible to uterine cancer tended more to receive HPV vaccine and the Pap tests to prevent cervical cancer [36, 37]. In another study, those with a higher perceived susceptibility showed a tendency to HPV vaccination 22.81 times as high as others [30]. In a review, Austin reported that low perceived susceptibility was a main barrier to administering the Pap test and breast self-administered test [38]. Contrary to the present findings, some other research showed that perceived susceptibility had no effect on the intention of receiving HPV vaccine in women [22, 39]. This divergence can be partly due to different demographic features of the participants, questionnaire content, sample size and statistical procedures. Researchers reckon that a certain level of perceived potential side effects of a disease is required before one is motivated to adopt a protective behavior [39]. Health specialists can report facts and figures about the infection at a national and international scale and, thus, increase women’s perceived susceptibility to GW.

As the present findings showed, perceived severity was positively correlated with the adoption of GW preventive behaviors. In other words, those with a higher perceived severity of GW adverse effects tended more to adopt protective behaviors. Theoretically speaking, Rosen and Stock (1974) maintained that perceived severity can increase preventive medical measures [40]. According to Bakhtiari, when someone perceives conditions critical, s/he tends to adopt a self-protective behavior [41]. In a similar vein, another study showed that women with a higher perceived fear of the mere thought of GW or cervical cancer and an increased heart rate agreed more than others with HPV vaccination [42]. Lee et al. maintained that perceiving cancer as a horrifying disease was the main reason for receiving HPV vaccines. As the participants with an intention of vaccination maintained, GW or uterine cancer can disrupt one’s work, education and even love affairs [43]. Similarly, in a number of other studies, perceived severity showed to have a significant effect on the rate of using HPV vaccination to prevent cervical cancer [30, 44]. Contrary to the present findings, another study found no significant effect of perceived severity on women’s intention of HPV vaccination [22]. This divergence can be partly explained by different demographic features, different questionnaire content, sample size and statistical methods.

The present researchers believe that knowledge, perceived susceptibility and severity together can encourage women to adopt healthy behaviors. As the present findings showed, knowledge, perceived susceptibility and severity were significantly correlated. Another study showed that knowledge managed to increase the rate of uterine cancer through affecting perceived severity [45]. It seems that if women have an adequate knowledge of GW preventive behaviors and symptoms and if they receive adequate and correct instructions with this regard, their perceived susceptibility to GW is increased. Thus, they tend more to perceive the severity of the disease and tend more to show preventive behaviors.

The present findings revealed that perceived benefits and barriers had no statistically significant correlation with GW preventive behaviors. Similarly, a number of studies showed no statistically significant correlation between perceived benefits and barriers and the intention of vaccination [34, 36, 46]. Contrary to the present findings, two other studies revealed the significant effect of perceived benefits and barriers on the intention of vaccination [30, 47]. These divergent findings can be partly explained by the different socio-demographic features of target research groups. In the majority of the above-mentioned studies, the aim was to screen for cervical cancer and receive HPV vaccines. However, in the present research, the aim of healthy behaviors was to prevent GW. It is noteworthy that though perceived benefits and barriers did not directly affect the target healthy behavior, path analysis showed the indirect effect of these constructs on the preventive behavior (moderated by perceived susceptibility and severity). These findings can be explained with several points in mind. Firstly, the questionnaire items probably did not reflect the participating women’s perceived benefits and barriers. Secondly, it seems that the participants did not perceive the benefits of adopting GW preventive behaviors. Probably more time is needed to adopt the former healthy behavior and perceive its benefits. It is also speculated when women do not perceive the benefits of a certain behavior, it indirectly affects their perception of barriers. This could have affected the statistically insignificant correlation between perceived benefits and barriers and GW preventive behaviors.

In the present findings, self-efficacy showed to be the strongest predictor of adopting GW preventive behaviors. Another study also confirmed this finding and introduced self-efficacy as the strongest predictor of the intention of HPV vaccination [34]. In another study, self-efficacy was found to be significantly correlated with the intention of HPV vaccination [48]. The correlation between self-efficacy and healthy behaviors was explored too and self-efficacy showed to be strongly affecting healthy behaviors. Someone with a low level of self-efficacy is less likely to try to adopt a healthy behavior or to change an already established habit [49]. Contrary to the present findings, in some other studies, self-efficacy did not show to affect the intention of HPV vaccination [50–52]. These differences can be partly due to participants’ different demographic features and purposes of research. We can say that people with a high self-efficacy have a greater knowledge, as according to TPB, behavioral success directly depends on perceived behavioral control and intention of healthy behavior if and only if an individual is very well aware of the current conditions [53].

## Conclusions

The present findings help to promote knowledge of the adoption of GWs preventive behaviors. In the light of the present findings we can conclude that HBM is a useful tool for health specialists as a theoretical framework for evaluating preventive programs and reducing sexually transmitted diseases such as GW preventive behaviors.

### Strengths and limitations

One strength of the present research was the large sample size as it included Bandar Abbas population. Still the generalization of findings is limited. Another strength of the present research was using HBM as the theoretical framework for adopting GW preventive behaviors among women. To our knowledge, this model had not been previously used in exploring GW preventive behaviors among women. The present findings can be used to develop educational interventions, focus on effective basic constructs to compare prospective studies in the field. There were certain limitations too. For one, the data were collected as self-reports. Probably, women considered social acceptance in their responses though the researcher tried to emphasize the confidentiality of information to reduce this bias to some extent.

Another limitation of the present research is the selection bias because there were chances that those living in the same neighborhood showed similar behaviors. We attempted to lower the selection bias by using a multi-stage sampling and randomization to maximize demographic variety. Still, it is recommended that in future a wider range of demographic features be used to include various women participants.

Furthermore, this research was cross-sectional in type and, thus, making causal claims is deemed less appropriate. Finally, as the questionnaires were completed only by women who were willing to participate in the research, the results cannot be representative of the perceptions of those who refused to take part in the research.

## Supplementary Information

**Additional file 1.** The data collection instrument.

**Additional file 2.** The data file.

## Data Availability

All data generated or analysed during this study are included in this published article and its Additional file 1 and Additional file 2

## Abbreviations

GW: Genital wart
HBM: Health belief model
SES: Socioeconomic status
