# Supplementary material for: A predictive model of genital warts preventive behaviors among women in the south of Iran: application of health belief model
Source: BMC Womens Health. 2022 Mar 8;22:63. doi: 10.1186/s12905-022-01649-6 (PMC8903721; doi:10.1186/s12905-022-01649-6)
Supplement: Supplementary file 1 — Additional file 1. The data collection instrument. [file 12905_2022_1649_MOESM1_ESM.docx]

**Respondent’s sociodemographic information**

**Age: .....................**

**Educational level:** elementary school ⃝ junior high school ⃝ high school ⃝ diploma ⃝ university ⃝

**Marital status:** Married Divorced widowed

**Occupation:** housewife ⃝ working at home ⃝ working outside home ⃝ retired ⃝

**Family size:** ....................

**Number of rooms in house:** ..................

**Insurance**: yes ⃝ no ⃝

**Knowledge of genital infection**

| item | statement | True | False | Don’t Know |
| --- | --- | --- | --- | --- |
| 1 | GWs virus is transmitted through sexual relationship. |  |  |  |
| 2 | GWs virus can be found in body fluids such as seminal fluid, on genital skin and surrounding areas as well as in mouth, throat and anus. |  |  |  |
| 3 | Condom can prevent GWs infection if it is used constantly. |  |  |  |
| 4 | It is impossible to eradicate GWs infection. |  |  |  |
| 5 | If a woman suspects infection in sex affair with husband or sex partner, she won’t need any examination or treatment anymore. |  |  |  |
| 6 | Blisters or severe itching of genitals, anus or surrounding areas can be a sign of GWs infection. |  |  |  |
| 7 | Unnatural secretions of women’s genital tract can be a sign of GWs. |  |  |  |
| 8 | Any change in genital secretions in smell, color and concentration can be a sign of GWs infection. |  |  |  |
| 9 | GWs infection can be asymptomatic. |  |  |  |
| 10 | GWs virus is not transmitted through sexual relationship. |  |  |  |
| 11 | One infected with GWs has the symptoms of infection. |  |  |  |
| 12 | There are more chances of infection with GWs in those who have sex with multiple partners. |  |  |  |
| 13 | GWs virus is a main cause of cervical cancer. |  |  |  |
| 14 | GWs virus might create ulcers in genitals. |  |  |  |
| 15 | Men might carry GWs infection. |  |  |  |
| 16 | GWs infection is not transmitted through physical (skin) contact. |  |  |  |
| 17 | An unnatural result of the Pap test can indicate GWs. |  |  |  |
| 18 | Genitals contact with mouth can cause warts and herpes and can transmit the GWs virus. |  |  |  |
| 19 | Wearing condom in all stages of sexual relationship does not reduce the chances of transmitting the virus. |  |  |  |
| 20 | There is no vaccine to prevent GWs virus. |  |  |  |
| 21 | GWs virus is often treated with antibiotics. |  |  |  |
| 22 | One might be infected with GWs without being aware of it. |  |  |  |
| 23 | Younger womenare at a lower risk of affliction with AIDS, herpes and GWs. |  |  |  |
| 24 | GWs infection during pregnancy or delivery cannot be transmitted to children. |  |  |  |

**Health Belief Model Questionnaire**

**“Perceived Susceptibility”**

| # | Rate the following statements for you. | Strongly agree | Agree | Neutral | Disagree | Strongly disagree |
| --- | --- | --- | --- | --- | --- | --- |
| 1 | I have more chances of affliction with GWs. |  |  |  |  |  |
| 2 | If my mother and sister are infected with GWs, I might get infected with the disease too. |  |  |  |  |  |
| 3 | As GWs infection is asymptomatic at the initial stages, I may be infected without any sign of the disease. |  |  |  |  |  |
| 4 | Despite adhering to genital health guidelines, I am at the risk of GWs infection. |  |  |  |  |  |
| 5  6 | Despite adherence to Islamic and familial rules and regulations, I am at the risk of GWs infection.  Women of a lower socioeconomic status stand more chances of GWs infection. |  |  |  |  |  |
| 7 | As I am a women, I am at the risk of GWs infection. |  |  |  |  |  |

**Perceived severity**

| # | Rate the following statements for you. | Strongly agree | Agree | Neutral | Disagree | Strongly disagree |
| --- | --- | --- | --- | --- | --- | --- |
| 1 | GWs infection affects family and job relations. |  |  |  |  |  |
| 2 | GWs infection can make me infertile. |  |  |  |  |  |
| 3 | GWs can lower my and my family’s spirits. |  |  |  |  |  |
| 4 | GWs, if not diagnosed and treated early, can lead to cervical cancer. |  |  |  |  |  |
| 5 | If I am infected with GWs, I may also get afflicted with other sexually transmitted diseases too. |  |  |  |  |  |

**Perceived benefits**

| # | Rate the following statements for you. | Strongly agree | Agree | Neutral | Disagree | Strongly disagree |
| --- | --- | --- | --- | --- | --- | --- |
| 1 | If I have the Pap test and am assured of my health, I develop a higher self-confidence. |  |  |  |  |  |
| 2 | A Pap test and early diagnosis of GWs can prevent high medical costs. |  |  |  |  |  |
| 3 | Adherence to health protocols is an economic way of preventing GWs. |  |  |  |  |  |
| 4 | A Pap test is a cost-effective way of lowering the risk of the disease and overcoming barriers to healthy behavior. |  |  |  |  |  |
| 5 | A Pap test is effective in preventing GWs. |  |  |  |  |  |
| 6 | One benefit of GWs vaccination is that it puts my mind at rest about sexual well-being. |  |  |  |  |  |
| 7 | GWs vaccination is effective preventing cervical cancer. |  |  |  |  |  |

**Perceived barriers**

| # | Rate the following statements for you. | Strongly agree | Agree | Neutral | Disagree | Strongly disagree |
| --- | --- | --- | --- | --- | --- | --- |
| 1 | A Pap test has a high cost. |  |  |  |  |  |
| 2 | Conducting a Pap test to diagnose GWs is painful. |  |  |  |  |  |
| 3 | Conducting a Pap test is hard and embarrassing to me. |  |  |  |  |  |
| 4 | I do not tend to have a Pap test as I am afraid of being diagnosed with a disease. |  |  |  |  |  |
| 5 | I feel embarrassed when others know I am infected with GWs. |  |  |  |  |  |
| 6 | The healthcare centers are crowded and not as helpful as expected. So, I prefer not to go there. |  |  |  |  |  |
| 7 | As I am employed, I hardly find time to go for a Pap test. |  |  |  |  |  |
| 8 | I am not willing to have a Pap test as the nurses in charge often misbehave. |  |  |  |  |  |
| 9 | I have not had a Pap test so far as I am not aware of its benefits. |  |  |  |  |  |
| 11 | I do not need a Pap test as I do not have a family history of uterine GWs. |  |  |  |  |  |

**Self-efficacy**

| # | Rate the following statements for you. | Strongly agree | Agree | Neutral | Disagree | Strongly disagree |
| --- | --- | --- | --- | --- | --- | --- |
| 1 | I take care of genital health issues to prevent infection with GWs. |  |  |  |  |  |
| 2 | I am sure I can wear condoms in all sex affairs. |  |  |  |  |  |
| 3 | I am sure I can overcome my fear of and embarrassment in having a Pap test. |  |  |  |  |  |
| 4 | I am sure I can find the time (despite much business) for the Pap test. |  |  |  |  |  |
| 5 | I am sure (despite financial problems) I can save enough money for a Pap test. |  |  |  |  |  |
| 6 | I am sure I can tolerate the little pain in the Pap test for the sake of my own health. |  |  |  |  |  |

**Healthy behavior**

| # | Rate the following statements for you. | Yes | No |
| --- | --- | --- | --- |
| 1 | I take a good care of genital health to lower the risk of infection with GWs. |  |  |
| 2 | I have the Pap test on a regular basis. |  |  |
| 3 | I wear a condom in every sex affair. |  |  |
| 4 | If a symptom of the disease emerges (e.g. vaginal bleeding or unnatural secretion), I visit a doctor ASAP. |  |  |
